# Adenosine in Acute Myocardial Infarction-Associated Reperfusion Injury: Does it Still Have a Role?

**DOI:** 10.3389/fphar.2022.856747

**Published:** 2022-05-13

**Authors:** Corrado De Marco, Thierry Charron, Guy Rousseau

**Affiliations:** ^1^ CIUSSS du Nord-de-l’Île-de-Montréal, Hôpital du Sacré-Coeur, Department of Medicine, QC, Montréal, Canada; ^2^ Department of Medicine, Université de Montréal, Montréal, QC, Canada; ^3^ Department of Pharmacology and Physiology, Université de Montréal, Montréal, QC, Canada

**Keywords:** myocardial infarction, no-reflow, reperfusion injury, adenosine, reperfusion, myocardial ischemia

## Abstract

The mainstay of acute myocardial infarction has long been timely reperfusion of the culprit obstruction. Reperfusion injury resulting from a multitude of pathophysiological processes has been demonstrated to negatively affect myocardial recovery and function post-infarction. Adenosine interacts directly with the sequential pathophysiological processes culminating in reperfusion injury by inhibiting them upstream. The evidence for adenosine’s benefit in acute myocardial infarction has produced mixed results with regards to myocardial salvage and long-term mortality. The heterogenous evidence with regards to benefits on clinical outcomes has resulted in modest uptake of adenosine in the clinical setting. However, it is critical to analyze the variability in study methodologies. The goal of this review is to evaluate how adenosine dose, route of administration, timing of administration, and site of administration play essential roles in the molecule’s efficacy. The benefits of adenosine, as highlighted in the following review, are clear and its role in the treatment of acute myocardial infarction should not be discounted

## Introduction

Reperfusion therapy for acute myocardial infarction (AMI) has been established as the mainstay of therapy for reduction of the extent of myocardial damage and, ultimately, of mortality (Fibrinolytic Therapy Trialists’ (FTT) Collaborative Group, 1994). Moreover, earlier reperfusion is related to improved survival (GUSTO Angiographic Investigators, 1993). That said, reperfusion itself has been associated with certain deleterious effects, including myocyte death, microvascular injury, myocardial stunning, no-reflow, and reperfusion arrhyhtmias (Kloner, 1993).

**FIGURE 1:**
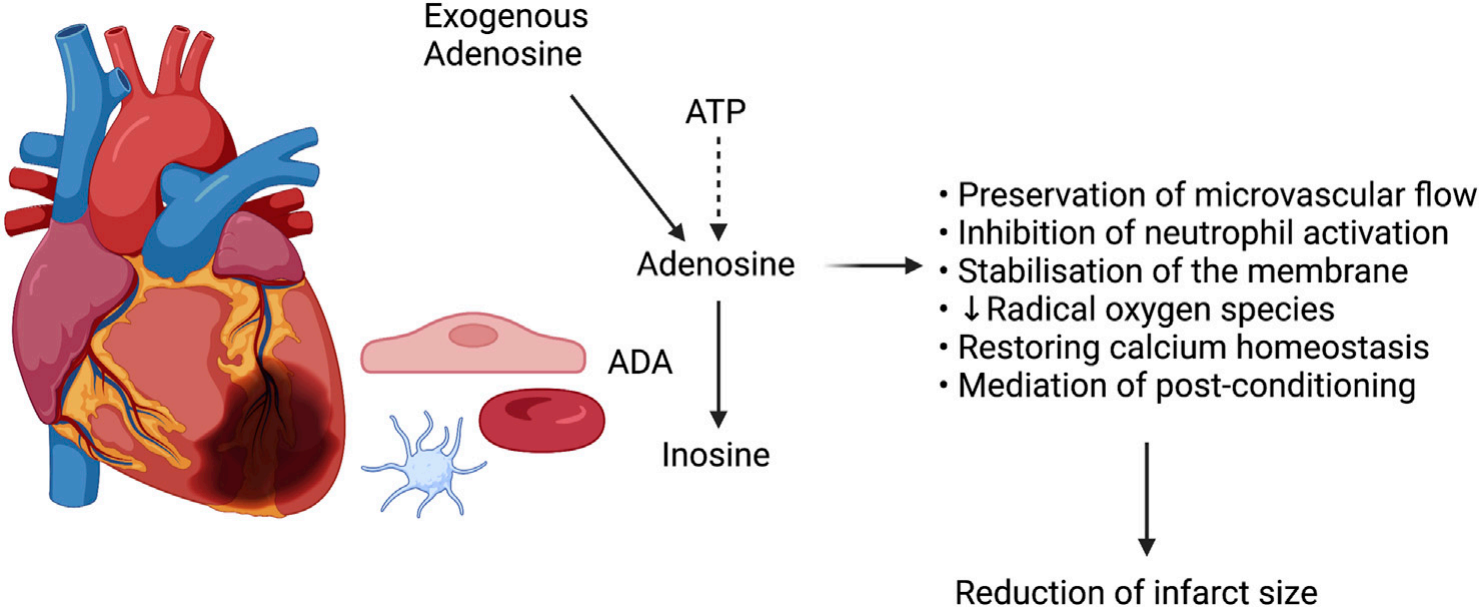
A schematic representation of adenosine’s protective role in myocardial ischemia.

Nowadays, the first-choice therapy for ST-elevation myocardial infarction (STEMI) is percutaneous coronary intervention (PCI) (Keeley et al., 2003). Although PCI is successful in most cases, up to 40% of patients do not achieve complete myocardial reperfusion despite successful angioplasty of the culprit lesion (Gao et al., 2015). Critically, the myocardial outcome of the “ischemia-reperfusion” sequence exhibited in AMI depends not only on the time delay between ischemia and reperfusion, but so too on whether lethal reperfusion injury occurs (Monassier, 2008a).

A multitude of therapeutic options aimed at minimizing reperfusion injury, and thereby optimising results and outcomes of coronary artery revascularization following AMI, have historically been proposed. Adenosine, an endogenous nucleoside produced by the degradation of adenosine triphosphate (ATP) (Forman et al., 2006), figures among them. Adenosine has been shown to act on all mechanisms of reperfusion injury, distal embolization excluded, by promoting preservation of microvascular flow, inhibiting neutrophils and the resultant inflammatory cascade, stabilizing cellular membranes and reducing synthesis of radical oxygen species, restoring calcium homeostasis, and mediating post-conditioning (Figure 1) (Forman et al., 2006; Monassier, 2008b).

Three large, randomised trials of adenosine completed in the late 1990s and early 2000s showed variable results. While adenosine consistently showed benefit in terms of minimizing infarct size, none of the studies were able to demonstrate a statistically significant mortality benefit, be it all-cause or cardiovascular mortality (Mahaffey et al., 1999; Quintana et al., 2003; Ross et al., 2005).

A systematic review published in 2015 (Gao et al., 2015) confirmed the same findings demonstrated in the three randomised control trials discussed above. Adenosine treatment was not found to have any effect on mortality or re-infarction, though did appear to offer beneficial effects on myocardial reperfusion, no reflow, and post-infarction left ventricular ejection fraction (Gao et al., 2015). Multiple hypotheses for the lack of consistent beneficial effects of adenosine have been emitted, including the inconsistent route of adenosine administration and the timing of infusion (Gao et al., 2015).

The conflicting findings in the literature, as well as the lack of statistically significant benefits in terms of endpoints pertaining to mortality and re-infarction, have resulted in sparse uptake in use of adenosine in the treatment of acute myocardial infarction.

## Revisiting the Concepts of Reperfusion Injury

*Monassier* succinctly summarizes the concept of reperfusion injury by separating it into its two main facets: no-reflow, resulting from endothelial microvascular injury, and reperfusion syndrome, involving cardiomyocytes (Monassier, 2008b). The no-reflow phenomenon was initially described in 1966 by Krug et al (Krug et al., 1966). This phenomenon results from microvascular dysfunction caused by increased capillary permeability and consequent edema in the infarcted/reperfused area, endothelial and vascular smooth muscle damage as well as release of vasoconstrictive substances from atherosclerotic lesions with resulted impaired vasomotion, and neutrophil infiltration and aggregation, all of which culminate in capillary destruction and hemorrhage (Heusch and Gersh, 2017; Heusch, 2019).

Consensus states that a large part of cell death resulting from reperfusion injury occurs within minutes of reperfusion. In addition to the mechanisms of microvascular dysfunction and vasoconstriction contributing to reperfusion injury, there seems to be an as-of-yet obscure but equally critical role mediated by the mitochondrial permeability transition pore (MPTP). Free radical oxygen species generation, which occurs during the ischemic event, and delivery to the infarcted area, which occurs following reperfusion, may contribute to MPTP opening. Opening of the MPTP appears to induce sarcolemmal rupture within the first minutes of reperfusion, potentially through the hypercontracture of neighbouring myocytes induced by high calcium levels in the presence of ATP (Komamura et al., 1994; Hausenloy et al., 2017; Heusch, 2020).

Reperfusion of the culprit lesion in AMI equally results in embolization of microthrombi and particles of plaque material downstream, thereby plugging small arteries and arterioles (Heusch and Gersh, 2017; Heusch, 2020; Kleinbongard and Heusch, 2022). These distal microinfarcts result in an inflammatory response and, moreover, reduce contractile function in the surviving myocardium around the distal microinfarcts via a mechanism of inflammatory signal-mediated myofibrillar oxidation (Kleinbongard and Heusch, 2022). Microvascular dysfunction in both the culprit artery, but so too in adjacent arteries via collateral vessels, may subsequently be induced by such embolic events (Heusch and Gersh, 2017; Heusch, 2020).

The processes above coalesce to ultimately contribute to abnormalities of potassium, sodium, and calcium homeostasis in injured myocytes, to an inability to restore myocyte energy balance, and to microvascular injury that are the hallmarks of the no-reflow phenomenon (Forman et al., 2006).

## Understanding Adenosine and the Rationale for its Use as a Cardioprotective Agent

Adenosine is a naturally occurring purine nucleoside comprised of an adenine molecule linked to a ribose sugar moiety via a beta-N9-glycosidic bond (Layland et al., 2014). It is synthesized in the intracellular space by purine synthesis or it may accumulate as a result of the breakdown of adenosine triphosphate (ATP) (Layland et al., 2014). Concentrations of intracellular adenosine have been shown to increase in situations in which there is a mismatch between ATP synthesis and use (e.g., ischemia) (Saito et al., 1999).

Adenosine’s transportation occurs rapidly into vascular endothelial cells and erythrocytes, where it is catabolized by adenosine deaminase to inosine (Layland et al., 2014). Notably, adenosine deaminase is found on the plasmatic membranes of erythrocytes and platelets (Franco et al., 1990). Understanding adenosine’s transportation and, most importantly, its short half-life is critical to comprehending its pharmacokinetics. Adenosine, when administered via the intra-coronary route, has a peak effect of < 10 s and a duration of action of roughly 20 s (Layland et al., 2014).

The four receptors that bind adenosine are A1, A2A, A2B, and A3 (Shryock and Belardinelli, 1997). Adenosine A2A receptors are found on neutrophils, endothelial cells, vascular smooth muscle, and platelets (Vinten-Johansen et al., 1999). Activation of the A2A and A2B receptors increases adenylate cyclase activity and cAMP levels via coupling to G_s_ proteins (Heusch, 2010), consequently resulting in potent vasodilation of the coronary circulation and an increase in myocardial blood flow (Shryock and Belardinelli, 1997; Vinten-Johansen et al., 1999; Heusch, 2010). Furthermore, adenylate cyclase activation *via* the A2A receptor results in inhibition of neutrophil superoxide generation and adherence to the endothelium (Vinten-Johansen et al., 1999). Therein, adenosine and its receptor-ligand induction of adenylate cyclase target neutrophil aggregation and adherence, reducing neutrophilic arterial plugging that has been evoked as one of the central mechanisms of reperfusion injury. Additionally, adenosine’s inhibition of neutrophil activation targets and prevents what is otherwise one of the initiating factors of the inflammatory cascade that culminates in reperfusion injury (Vinten-Johansen et al., 1999).

Adenosine’s administration initiates a multitude of metabolic events that could be beneficial in the setting of ischemia and reperfusion (Forman et al., 2006). Exogenous adenosine is responsible for the restoration of ATP levels in viable but energy-deficient ischemic cells, as demonstrated in a study wherein the presence of an adenosine deaminase inhibitor resulted increased levels of ATP following reperfusion (Forman and Velasco, 1991).

Studies suggest that adenosine may play a critical role in promoting vascular repair and in accelerating the development of new blood vessels following vascular injury (Dubey et al., 2002; Montesinos et al., 2004). These properties of adenosine are attributable to its stimulation of angiogenic factors such as IL-8, vascular endothelial growth factor, and basic fibroblast growth factor from microvascular endothelial cells (Feoktistov et al., 2003). One may infer from the above properties that adenosine may play an integral role in the repair of injured endothelium after reperfusion and, thereby, prevent ventricular remodeling by favouring collateral blood flow.

Ischemic pre-conditioning is a phenomenon wherein a brief period of ischemia renders the myocardium more resistant to injury following a subsequent episode of ischemia (Vinten-Johansen et al., 1999). Seminal work done by Schulz et al (Schulz et al., 1995) has highlighted the integral role that endogenous release of adenosine plays in limitation of infarct size. The group’s work (Schulz et al., 1995), published in 1994, compared four groups: a first group subjected to 90 min of ischemia, a second group subjected to an initial 10-minute period of ischemia (i.e., ischemic pre-conditioning) followed by a 15-minute period of reperfusion prior to a 90-minute period of ischemia, a third group receiving intra-coronary infusion of adenosine deaminase maintained throughout the 90-minute ischemic period, and a final group undergoing ischemic pre-conditioning with concomitant adenosine deaminase infusion and subsequent 90-minute ischemia. Their results demonstrated a significantly reduced infarct size in the ischemic pre-conditioning alone (i.e., without adenosine deaminase infusion) group (Schulz et al., 1995). In so doing, this pioneering article confirmed the central role of endogenous adenosine in ischemic pre-conditioning in swine hearts, since the infarct size reduction in ischemic pre-conditioning was significantly attenuated when catabolism of adenosine was increased through administration of adenosine deaminase (Schulz et al., 1995). A similar principle, dubbed ischemic post-conditioning, is a phenomenon whereby repetitive short episodes of ischemia following a longer, more prolonged ischemic episode result in significantly decreased myocardial damage (Liu et al., 1991; Zhao et al., 2003). Adenosine, *via* A1 receptor-induced activation of protein kinase C and subsequent protein function modulation by phosphorylation mediates and potentiates the phenomena of pre- and post-conditioning (Vinten-Johansen et al., 1999). Lending further credence to this theory, research demonstrated that pre-conditioning protection could be blocked by use of adenosine receptor blocking agents (Liu et al., 1991; Schulz et al., 1995), and was conversely potentiated by use of an A1-specific agonist (Liu et al., 1991). The A2A and A3 receptors may equally play a role in pre- and post-conditioning, though this has been less well established.

Moreover, the ischemic pre-conditioning triggered by adenosine has been demonstrated to be unique when compared to that triggered by other molecules mediating pre-conditioning, such as acetylcholine, bradykinin, and opioids (Cohen et al., 2001). More specifically, it has been demonstrated that the pre-conditioning induced by adenosine release, unlike that of the other molecules mentioned, seems independent of either free radicals or mitochondrial K_ATP_ channels (Cohen et al., 2001). The evidence supports that it is therefore most likely that adenosine results in direct activation of protein kinases (Cohen et al., 2001), more specifically protein kinase C, whose role in ischemic pre-conditioning is well established (Vinten-Johansen et al., 1999).

The beneficial effects of adenosine on myocardial blood flow and on limitation of infarct size have been extensively studied and demonstrated in animals (Boucher et al., 2004; Yetgin et al., 2015).

## Highlighting the Importance of the Administration Route

Early studies of adenosine in the setting of AMI in humans were performed with administration of adenosine *via* the intravenous route.

In the AMISTAD (Mahaffey et al., 1999; Ross et al., 2005) trials, low (50 mcg/kg/min) and high (70 mcg/kg/min) infusions of adenosine started within 15 min of thrombolysis or before coronary intervention were compared in a 1:1:1 fashion to placebo. AMISTAD II demonstrated no difference in the primary endpoint of new congestive heart failure > 24 h after randomization or first re-hospitalization for congestive heart failure, or death from any cause within 6 months, but that patients treated with adenosine had a tendency towards smaller infarct sizes (17% of the left ventricular size versus 27%, *p* = 0.074). Furthermore, infarct size was shown to be significantly related (*p* < 0.001) to the occurrence of the primary endpoint.

In the ATTAC (Quintana et al., 2003) study, a single dose (10mcg/kg/min) of adenosine was infused at the time of thrombolysis and maintained for 6 h and was compared to placebo. No beneficial effect of adenosine was found regarding echocardiographic indices of left ventricular systolic or diastolic function. Despite the trial being stopped early due to lack of apparent effect after an interim analysis, 12-month follow-up data showed that cardiovascular mortality was 8.9% with adenosine and 12.1% with placebo (OR 0.71, 95% CI 0.4-1.2, *p* = 0.2) and 8.4 versus 14.6% (OR 0.53, 95% CI 0.23-1.24, *p* = 0.09) in patients with anterior AMI.

The primary limitation pertaining to intravenous adenosine administration is that maximal doses are difficult to achieve because of the marked systemic hypotension associated with higher adenosine doses (Charron et al., 2013). Whether maximal doses of adenosine were achieved in studies employing the intravenous route is unclear. The cardioprotective properties observed, however, are non-negligible and highlight that even intravenous adenosine, despite potentially sub-optimal dosing, may result in stimulation of remote pathways of cardioprotection. Intracoronary administration of adenosine, unlike intravenous administration, enables delivery of much higher doses without direct systemic effects, most notably hypotension.

## Properly Timing Adenosine Administration

Recalling that the pathophysiology underpinning reperfusion injury is, as the name implies, coronary reperfusion, it is inherently logical that the optimal timing for adenosine administration be prior to reperfusion, whether achieved with thrombolysis or percutaneous coronary intervention. If administered late, once reperfusion has already occurred, the pathophysiological cascade culminating in reperfusion injury will already have been triggered, and adenosine’s inhibitory effects on this chain of events are more likely to be inefficient.

To the above point, an animal study on rats published in 2004 (Boucher et al., 2004) compared the effects of early versus late administration of an adenosine A2A agonist (*CGS21680*) on infarct size, which was calculated as a percentage of the area at risk by occlusion of the target coronary artery—in this case, the left anterior descending artery. In the study, the early administration group received *CGS21680* 5 min prior to reperfusion, whereas the late administration group received adenosine 5 min after the initiation of reperfusion. Study groups were compared to control subjects. Infarct size was significantly reduced in the early compared to the control group (25.7 ± 5.3% versus 46.5 ± 5.3%, *p* < 0.05). Conversely, there was no statistically significant difference when comparing the late group infarct size area (38.2 ± 6.2%) to that of the control group. Indeed, such a study lends credence to the belief that timing adenosine administration before reperfusion is crucial for optimising treatment effects.

To date, there has been inconsistency in study methodology as it pertains to adenosine administration (Yetgin et al., 2015). Ultimately, results have been mixed, though there is a demonstrable trend towards improved outcomes in terms of infarct size limitation with earlier administration of adenosine.

## Establishing an Optimal Duration of Adenosine Infusion

Duration of infusion represents yet another point of discussion when examining adenosine’s role in AMI. The early intravenous studies (Mahaffey et al., 1999; Quintana et al., 2003; Ross et al., 2005), most notably AMISTAD, AMISTAD II, and ATTACC, used variable infusion durations ranging from one to six h. That said, the limitations of intravenous adenosine administration have been discussed previously and, indeed, dose adjustments to infusion rates were often made in these studies because of adverse effects related to the adenosine infusion.

In contrast, while there may be no human studies evaluating continuous infusions of adenosine *via* the intracoronary route, an animal study on porcine subjects (Yetgin et al., 2015) did compare bolus with prolonged infusions of intracoronary adenosine. Results of said study (Yetgin et al., 2015) demonstrated that only the prolonged, high-dose infusion of adenosine was able to limit infarct size (46 ± 4% of the area at risk versus 59 ± 3% in the control group, *p* = 0.02) and no reflow (26 ± 6% of the infarct area versus 49 ± 6% in the control group, *p* = 0.03), with bolus administration of adenosine without subsequent prolonged infusion yielding results that were similar to those of the control group.

Given the extremely short half-life of adenosine, it stands to reason that bolus injections, unless perfectly timed and optimally delivered, may be inadequate when treating reperfusion injury in AMI. Conversely, more prolonged intracoronary delivery initiated just prior to reperfusion may increase local drug concentrations multi-fold and may achieve adequate levels in the target microvascular bed, thereby potentially improving therapeutic efficacy. That said, There remains at present a lack of evidence in the literature to support this hypothesis.

## THE Importance of Location of Adenosine Administration

The logical question stemming from growing interest in intracoronary administration of adenosine pertains to selection of the optimal location for such intracoronary delivery. Studies to date have employed various methodologies, with intracoronary injection sites being quite variable. The three main intracoronary administration sites identified and examined in the literature thus far are at the catheter site, at the site of percutaneous coronary intervention, or distal to the occlusion site (Yetgin et al., 2015).

An animal study (Charron et al., 2013) in male pigs harmonizes many of the elements discussed above by comparing three different doses of adenosine—2, 4, and 8 mg—administered prior to reperfusion as a bolus either intra-coronary, downstream of the occlusion site, or extra-coronary. Results demonstrated a significant reduction (*p* < 0.05) in infarct size expressed as a percentage of area at risk in the 4 mg (33 ± 6%) and 8 mg (30 ± 5%) intra-coronary groups compared to the placebo group (46 ± 3%). Critically, there was no statistically significant reduction in the 2 mg intra-coronary group (38 ± 2%) and, most interestingly, in the 8 mg intracoronary group in whom adenosine was injected upstream of the occlusion site (47 ± 6%).

Translation of such animal studies to humans, with the beneficial effects of intra-coronary adenosine downstream of the occlusion site prior to the onset of reperfusion, has been demonstrated by Marzilli et al. (Marzilli et al., 2000). Such an administration route was associated with beneficial effects on both coronary blood flow and ventricular function, indicating likely minimization of the mechanisms underpinning reperfusion injury (Marzilli et al., 2000). In fact, a meta-analysis comparing randomized controlled trials studying intracoronary adenosine versus control to trials studying intravenous adenosine versus control found improved clinical outcomes in terns of less heart failure in patients administered intracoronary adenosine (Bulluck et al., 2016).

## Discussion

The mainstay of therapy for patients with AMI remains reperfusion, either *via* timely percutaneous coronary intervention in capable centers or *via* thrombolysis. Successful reperfusion has been extensively demonstrated to reduce cardiac mortality and minimize infarct size and left ventricular damage. That said, reperfusion, as discussed extensively in this review, may itself induce injury deleterious to left ventricular function both in the short- and long-term. Therefore, exploration and identification of therapy tailored to those at the highest risk of reperfusion injury may provide additional avenues dedicated to improving left ventricular function and clinical outcomes.

Adenosine has widely been demonstrated to have multiple favorable effects in mitigating no-reflow and reperfusion injury, primarily but not exclusively through inhibition of neutrophil-mediated vascular plugging and vascular damage. In experimental models, both intravenous and intracoronary routes of administration resulted in marked reduction of infarct size. In human trials, intravenous adenosine has similarly shown benefit in infarct size reduction, though this benefit has yet to translate to concrete clinical endpoints, most notably cardiovascular mortality and heart failure hospitalization.

While use of adenosine in the acute treatment of AMI has yet to garner uptake at large, it remains an avenue of interest worth exploring further. Most critically, the varying study methodologies make it difficult to draw broad conclusions from the existing evidence. The evidence is becoming increasingly clearer that dose, timing of administration, route of administration, and site of administration play critical roles in adenosine’s ultimate efficacy. An understanding of adenosine’s pharmacokinetics and pharmacodynamics is critical when examining studies done to date and when planning and executing future studies. In the case of intra-coronary administration, especially, the presence of blood in the catheter may lead to rapid inactivation of adenosine, recalling that adenosine deaminase is found on the plasmatic membrane of erythrocytes and platelets, prior to its arrival at the target site. Overall, by drawing from the literature, it appears as though a bolus of adenosine delivered intra-coronary distal to the occlusion site at or just prior to reperfusion is most effective in reducing reperfusion injury, though this hypothesis has yet to be tested in human subjects.

## Conclusion

It is critical that reperfusion injury be seen not only as a theoretical phenomenon, but rather as an important determinant of final infarct size and consequent ventricular dysfunction. Adenosine, despite its perceived lack of benefit relative to clinical outcomes, remains one of the few molecules to consistently reduce infarct size, especially in anterior AMI, by directly combatting the pathophysiological underpinnings of reperfusion injury. Its clinical use and continued study in well-designed experiments is not only necessary, but so too vital to the goal of improving patient outcomes in AMI.
